# The American college of radiology diagnostic fluoroscopy dose index registry pilot: Dosimetric performance and benchmarking challenges

**DOI:** 10.1002/acm2.70458

**Published:** 2026-01-26

**Authors:** Steve D. Mann, Donald L. Miller, Grant Fong, Allen R. Goode, Emily L. Marshall, Thomas Nishino, Pavlina Boxx, Liqiang Ren, Celalettin Topbas, Alan H. Schoenfeld, Vivek Singh, Jie Zhang

**Affiliations:** ^1^ Department of Radiology Duke University Durham North Carolina USA; ^2^ US Food and Drug Administration Silver Spring Maryland USA; ^3^ Imaging, Diagnostic Institute Cleveland Clinic Foundation Beachwood Ohio USA; ^4^ Department of Radiology and Medical Imaging UVA Health Charlottesville Virginia USA; ^5^ Department of Radiology University of Chicago Chicago Illinois USA; ^6^ Division of Medical Physics University of Florida Gainesville Florida USA; ^7^ Department of Imaging Physics The University of Texas MD Anderson Cancer Center Houston Texas USA; ^8^ Radiation Safety Department Huntsville Hospital Huntsville Alabama USA; ^9^ Department of Radiology UT Southwestern Medical Center Dallas Texas USA; ^10^ Department of Radiology Montefiore Medical Center Bronx New York USA; ^11^ Department of Radiology University of Kentucky College of Medicine Lexington Kentucky USA

**Keywords:** Dose Index Registry, Fluoroscopy, Radiation Dose Monitoring, Radiation Dose Structured Report

## Abstract

**Background:**

The ACR Diagnostic Fluoroscopy Dose Index Registry (DIR‐Fluoro) is expanding to include diagnostic fluoroscopy. Variations in dose reference points and overhead radiography events may introduce unique challenges for benchmarking.

**Purpose:**

To survey the technological status and dosimetric performance of fluoroscopes participating in the DIR‐Fluoro pilot project, focusing on longitudinal stability and variability of fluoroscopic dose reporting accuracy across multiple institutions and vendors.

**Methods:**

Sixty‐six fluoroscopic systems from nine institutions (24 facilities) were surveyed for facility type, fluoroscope type, image receptor type, age, dose reporting capabilities, and other key features. Of these, 56 were evaluable. Semi‐annual measurements assessed reference air kerma (K_a,r_) and air kerma area product (P_KA_) accuracy. Linear mixed‐effects models evaluated changes in dose accuracy over time, incorporating system‐specific random effects; models were compared using likelihood ratio testing. Radiation Dose Structured Reports (RDSR) contents were investigated to understand the challenges in benchmarking diagnostic fluoroscopy dose indices.

**Results:**

Nearly 80% of units were tube‐under‐table fluoroscopes. Average age was 9.6 ± 5.2 years. Sixty‐four percent of the units produced RDSRs. Median deviations for K_a,r_ and P_KA_ were 1%–4%. Accuracy of P_KA_ and K_a,r_ remained stable, with no significant time‐dependent drift for RDSR‐capable systems (*p* > 0.05). Incorporating detector type significantly improved performance for P_KA_ measurements (*p* < 0.05 for all datasets); K_a,r_ models were generally best fit by simpler models (*p* > 0.05 for 3 of 4 datasets). Major discrepancies in RDSRs were observed, including differences in K_a,r_ reference point definitions and in event‐level data. Overhead radiography exposures were not well distinguished from fluoroscope exposures. These issues resulted in inconsistencies in reported K_a,r_ values.

**Conclusion:**

Fluoroscopic dose indices were accurate and stable over time. Differences in RDSR availability result in data biased to newer systems with flat panel detectors. Discrepancies in RDSR content and inconsistent reference point definitions necessitate use of P_KA_ as the primary benchmark metric.

## 1 INTRODUCTION

The American College of Radiology (ACR) houses various data registries under the National Radiology Data Registry (NRDR). Among these is the Dose Index Registry (DIR), including modules for the CT registry (DIR‐CT) and the more recent interventional fluoroscopy registry (DIR‐Fluoro).^1^, ^2^ The DIR provides a means for facilities to benchmark dosimetry data for various imaging modalities used in radiology procedures. Benchmarking allows for a comparison of readily available dose metrics to aid facilities in optimizing imaging protocols, managing patient radiation exposure, and identifying outliers which present opportunities for clinical improvements. Benchmarking and protocol review for CT is also expected by some accrediting bodies, such as The Joint Commission,^3^ and many facilities utilize the DIR‐CT to aid in meeting such standards.

In 2018, the ACR launched the first stage of the DIR‐Fluoro with a focus on interventional procedures. Interventional procedures were chosen due to the relatively wide availability of Radiation Dose Structured Reports (RDSRs) used for the collection of dosimetry data in fluoroscopes designed for use in interventional procedures. The pilot phase established the basic workflow for participants, including mapping methodologies, user dashboards, and key metrics for benchmarking. The study included semi‐annual dose index verification for interventional fluoroscopes, which both characterized the technical elements related to dosimetry and provided an overview of the equipment and facility characteristics sampled during the pilot phase. At completion of the pilot phase in 2020, the DIR‐Fluoro for interventional procedures officially launched for all DIR participants. Studies published on the pilot results showcased both performance surveys and procedure dosimetry along with a comparison to the RAD‐IR study from the early 2000s.^2^, ^4^, ^5^, ^6^, ^7^

In 2022, the next phase of the DIR‐Fluoro launched with a focus on diagnostic fluoroscopy procedures. This phase aimed to establish the framework for benchmarking radiation dose from diagnostic fluoroscopy procedures. From a technical standpoint, benchmarking for diagnostic fluoroscopy procedures is more complicated than for interventional procedures for many reasons, including the diversity of fluoroscopes used, limitations of RDSR availability and contents, and frequent inclusion of exposure from radiographic (overhead) x‐ray tubes as part of fluoroscopic studies.

This publication presents the first stage of the DIR‐Fluoro diagnostic fluoroscopy pilot with the aim of characterizing the technical characteristics of participating fluoroscopes. Fluoroscope evaluations include dosimetric accuracy and consistency, dose reporting limitations, fluoroscope age, image receptor types, and other system and operational characteristics. Understanding the heterogeneity of fluoroscopes used for diagnostic fluoroscopy procedures is necessary to establish tools for users of the DIR‐Fluoro and to understand the limitations of benchmarking for these procedures.

## 2 MATERIALS AND METHODS

Twenty‐four facilities across nine corporate accounts participated in the DIR‐Fluoro diagnostic pilot. The participating institutions included: The Cleveland Clinic, Duke Health, Huntsville Hospital Health System, Montefiore Medical Center, University of Chicago Medical Center, University of Kentucky Healthcare, University of Texas MD Anderson Cancer Center, University of Texas Southwestern Medical Center, and University of Virginia Health. Table 1 summarizes the types and populations served of the participating facilities at each institution.

**TABLE 1:** Summary of characteristics of participating corporate accounts and their facilities in the DIR‐Fluoro diagnostic pilot.

**Number of corporate accounts**:	9
**Types of facilities within corporate accounts**:	
Academic/University hospitals:	11
* Community hospitals*	13
**Facility populations served**:	
* Metropolitan (> 100k)*	15
* Small/Suburban (50–100k)*	8
* Rural (< 50k)*	1

Fluoroscopes at participating facilities were surveyed semi‐annually over a two‐year period (2022–2023). Sixty‐six fluoroscopes were surveyed during the pilot study (Table 2), including a mix of tube‐under‐table, tube‐over‐table, and C‐arm units (both fixed (multi‐purpose) and mobile, but not including mini C‐arms); no interventional fluoroscopes were included in the study. Surveys were used to collect information on fluoroscope characteristics and technical information, information related to operator groups and personnel shielding availability, and semi‐annual measurements of reference point air kerma (K_a,r_) and air kerma area product (P_KA_) accuracy, following the methods of AAPM TG 190.^8^ Both K_a,r_ and P_KA_ were measured independently for fluoroscopic and radiographic acquisition modes of operation with the fluoroscopy x‐ray tube. The average of three measurement runs for each mode, with a target of approximately 50 mGy for each series, was used to determine accuracy of the displayed values. Overhead radiograph acquisitions were not included. Data were collected using an Excel template (Microsoft, Redmond, Washington) containing both a questionnaire and measurement guide for each Spring and Fall of each year of the study. Deviations (percent errors) were calculated for each metric to determine the accuracy of the fluoroscope reported values. Deviation is calculated as:

$$\mathrm{Deviation}\;=\frac{\left(\mathrm{Metri}c_{\mathrm{Displayed}}-\mathrm{Metri}c_{\mathrm{Measured}}\right)}{\mathrm{Metri}c_{\mathrm{Measured}}}\;\times 100\%$$

**TABLE 2:** Distribution of vendors and types of fluoroscopes participating in the DIR pilot.

Manufacturer	Tube‐under‐table systems	Tube‐over‐table systems	Fixed C‐arms	Mobile C‐arms
Siemens	31	4	5	0
GE	5	0	0	1
Philips	16	0	3	0
Canon	0	0	1	0

The characteristics and technical information requested included information about the fluoroscope manufacturer, model, system type, detector type, displayed dose metrics, and the ability of the system to produce Radiation Dose Structured Reports (RDSR) or other alternative dose summaries. Additional ancillary operation information for potential use in other aspects of the pilot included the availability of pulsed fluoroscopy, number of in‐room monitors, and whether facilities have trainees performing procedures regularly. For systems producing RDSRs, example RDSRs were individually examined to identify potential concerns that might impact registry data collection.

Reported K_a,r_ and P_KA_ values were analyzed to examine compliance with U.S. regulatory limits and international standards (± 35%)^9^, ^10^ and consistency of performance (both individually and collectively) during the study duration. Both indices were evaluated separately for fluoroscopic and acquisition modes. Linear mixed‐effects (LME) models were fit to evaluate the fixed effect of time (to identify changes in accuracy during the pilot) while accounting for repeated measurements from individual systems using random intercepts and, where appropriate, random slopes. Additional models included detector type, flat panel detector (FPD) versus image intensifier (II), as a fixed effect. Given the requirement for RDSRs for facilities to participate in the DIR, separate analyses were conducted limited to systems with RDSR capability. Table 3 summarizes the four models that were evaluated: a time‐only model with random intercepts (Model 1) and models incorporating detector type (Model 2), random slopes for time (Model 3), and both (Model 4). Detector type served both as a proxy for overall system technology and as a factor that may influence dosimetric differences, particularly for circular field‐of‐view systems. Random slopes allow for individual system trends to be modeled. Random intercepts account for the potential existence of baseline differences among individual systems. Likelihood ratio tests were used to compare nested models by evaluating the difference in log‐likelihoods, with significance assessed using a chi‐squared distribution. All dosimetry data analysis and modeling were performed using MATLAB 2022b (Mathworks, Natick, Massachusetts).

**TABLE 3:** Summary of LME Models used in dosimetric stability analysis.

LME Model:	Description:
Model 1	Time only (random intercept)
Model 2	Time + Detector Type (random intercept)
Model 3	Time only (random intercept and slope)
Model 4	Time + Detector Type (random intercept and slope)

## 3 RESULTS

**FIGURE 1:**
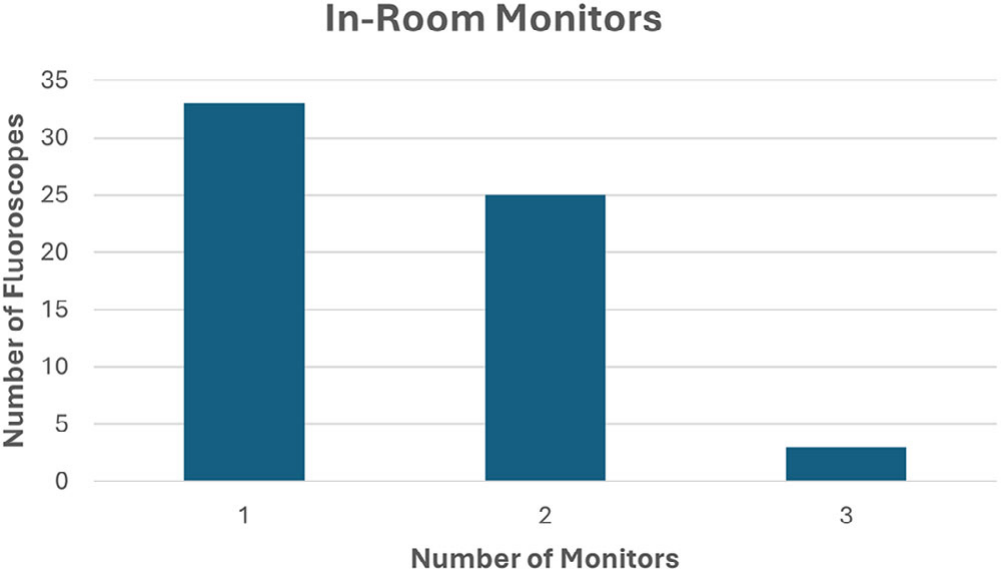
Number of in‐room monitors reported for each fluoroscope from survey.

**FIGURE 2:**
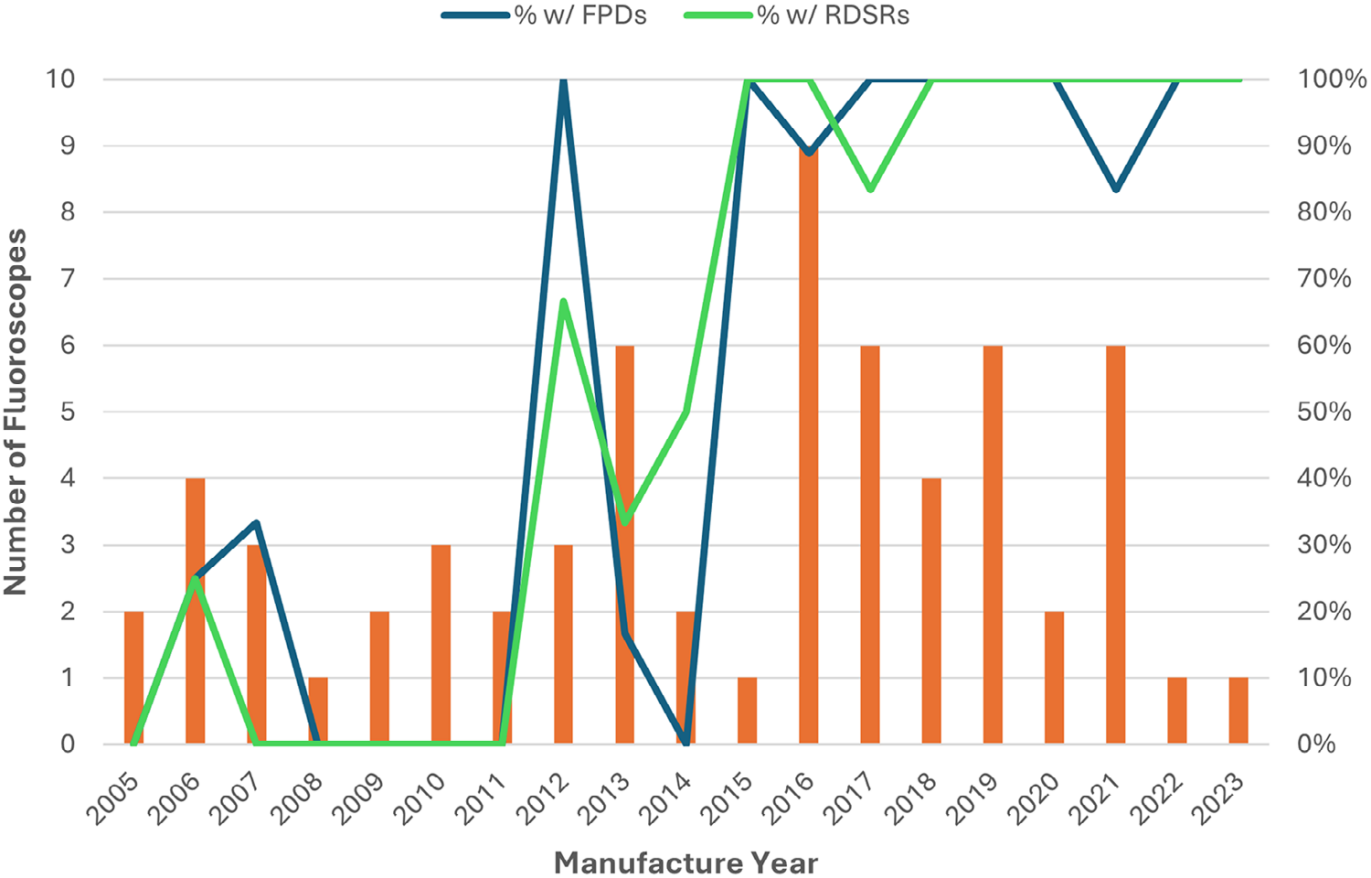
Plot showing the number of fluoroscopes manufactured each year among those participating in the DIR pilot. The percentages of all fluoroscopes manufactured in the given year with a Flat Panel Detector (FPD) (blue line) and with Radiation Dose Structured Report (RDSR)‐capability (green line) are also plotted.

A summary of results from the characteristics and technical information survey is shown in Table 4, while Figure 1 shows a histogram of surveyed systems according to the number of available in‐room monitors for the fluoroscope. Systems with multiple monitors offer operators the ability to view more than one image series simultaneously during procedures, which may impact overall radiation utilization. Figure 2 illustrates the strong correlation between detector type, age, and RDSR capability. In general, fluoroscopes in the pilot manufactured since 2015 have been mostly equipped with FPDs and nearly all have RDSR capabilities.

**TABLE 4:** Summary of system and operational characteristics among those surveyed in the DIR pilot.

**Detector type**:	
*Flat panel detectors*	62%
*Image intensifiers*	38%
**Average system age**:	9.6 ± 5.2 years
**Produces RDSR**	64%
**Produces alternative dose summary**	50%
**Dose indices available**:	
*K_a,r_*	93%
*K_a,r_ rate*	92%
*P_KA_*	98%
**Pulsed fluoro available**:	97%
**Trainees perform procedures**:	78%

Periodic verification of K_a,r_ and P_KA_ was performed on 56 of 66 surveyed fluoroscopes. Limitations included a combination of availability (e.g., remote sites) or system age (no displayed K_a,r_ and P_KA_ values). Sites were encouraged to submit the characteristics and technical information survey for these units despite inability to report dose indices to capture the state of technology among the pilot sites. Within the cohort collecting dose data, the number of measurements varied according to both when the site joined the pilot project and how long the system was in operation (due to repair, removal, or replacement). In total, 170 dose index verification datasets for fluoroscopic and radiographic acquisition modes of operation were collected during the 2‐year study period.

**FIGURE 3:**
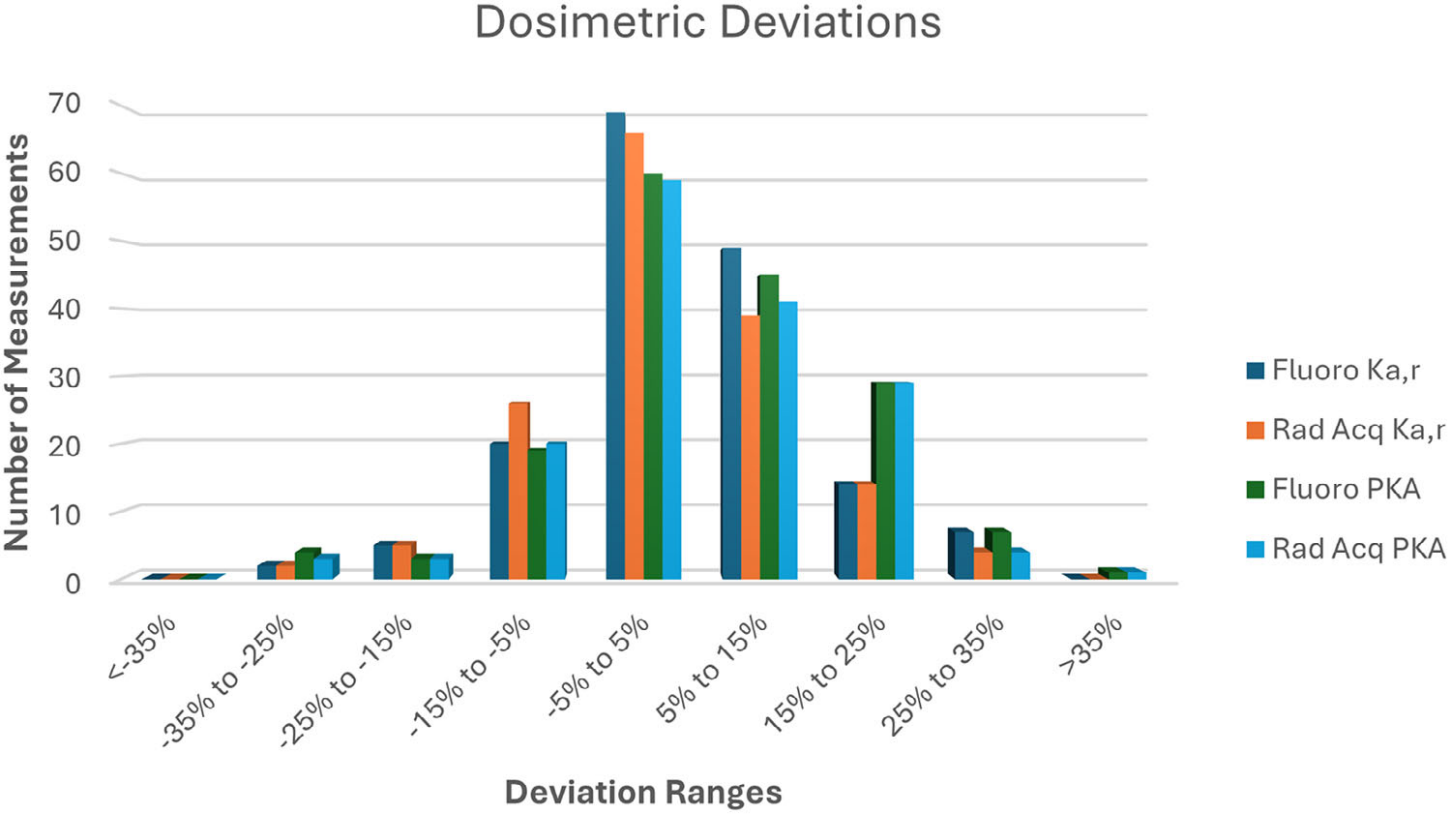
Histogram of dosimetric deviations for each of the dose indices tracked during the DIR pilot. Larger magnitude values represent greater accuracy errors in the displayed dose indices.

Figure 3 plots the measured versus displayed deviations in K_a,r_ and PKA values, separated by fluoroscopic and acquisition modes. Values near zero correspond to high accuracy of the fluoroscope‐reported dose metrics when compared to direct measurements.

Median deviations of fluoroscopy dose indices were 3% and 4% for K_a,r_ and P_KA_. For radiographic acquisition mode of operation, median deviations were 1% and 4% for K_a,r_ and P_KA_. Only two measurements (fluoroscopy and radiographic acquisition P_KA_) for one system fell outside the required ± 35% accuracy range during one of the semi‐annual surveys.

Results of the linear mixed effects model are shown in Tables 5 and 6. Likelihood ratio testing showed that among the complete datasets (Fluoroscopy Mode K_a,r_, Fluoroscopy Mode P_KA_, Acquisition Mode K_a,r_, Acquisition Mode P_KA_), the models for P_KA_ measurement for both Fluoroscopy and Acquisition were improved by the addition of detector type and to a lesser extent random slopes (*p* < 0.05). However, for K_a,r_, models that included detector type or random slopes did not significantly improve the model fit compared to the simpler time‐only model (*p* > 0.05). When restricted to only RDSR‐capable systems, Model 4 and Model 2, which both include detector type, were more frequently identified as the best model.

**TABLE 5:** Summary of linear mixed effect model comparisons for the full dataset.

Mode of operation	Best model	Model *p* (Best model vs model1)	LR statistic	Time *p* in best model
Fluoroscopy K_a,r_	Model1	–	–	0.36
Fluoroscopy P_KA_	Model4	<0.001	43.9	0.02
Acquisition K_a,r_	Model1	–	–	0.44
Acquisition P_KA_	Model4	0.02	20.9	0.06

**TABLE 6:** Summary of linear mixed effect model comparisons for only RDSR‐capable systems.

Mode of operation	Best model	Model *p* (Best model vs model1)	LR statistic	Time *p* in best model
Fluoroscopy K_a,r_	Model4	0.046	18.6	0.48
Fluoroscopy P_KA_	Model4	<0.001	30.5	0.12
Acquisition K_a,r_	Model1	–	–	0.43
Acquisition P_KA_	Model2	0.01	6.4	0.06

Within the models, the variable ‘time’ quantified the tendency for the deviation values to change over the course of the pilot period. Time was generally found to be not significant for both the simplest model (Model 1) and (where applicable) the best model from Tables 5 and 6 suggesting stable deviation values during the DIR pilot period. Only Fluoroscopy P_KA_ measurements comprising the entire dataset demonstrated a significant time dependence. For only RDSR‐capable systems, time was not found to be a significant variable in any model.

### 3.1 RDSR review findings

As part of the DIR‐FL pilot, RDSRs were reviewed from participating fluoroscopy systems to evaluate consistency and reliability for dose monitoring. Evaluations across fluoroscopy systems revealed multiple challenges and discrepancies in how the Patient Entrance Reference Point (PERP) was defined and applied.

#### 3.1.1 Inconsistent reference point definitions

Some systems listed multiple or ambiguous Patient Entrance Reference Point (PERP) locations for accumulated K_a,r_. For example, one system specified three separate PERP positions (“1 cm above Tabletop,” “30 cm above Tabletop,” and “30 cm in front of the image input surface”). Another system, despite being a tube‐under‐table configuration with no isocenter, reported the reference point as “15 cm from the isocenter toward source.” A few systems reported the PERP as “30 cm above Tabletop and 30 cm in Front of Image Input Surface” for the single value of accumulated K_a,r_.

#### 3.1.2 Mismatch between event and accumulated metrics

Several systems reported ambiguous or nonsensical PERPs at the individual radiation event level as well. This included event‐level PERPs defined relative to isocenter for the same tube‐under‐table unit mentioned previously, including both fluoroscope and overhead radiography exposures. Another system reported all irradiation events to occur with a PERP “30 cm above the Tabletop” despite the events being a mixture of tube‐under‐table fluoroscope exposures and overhead radiography exposures.

#### 3.1.3 Flawed summing of, or inconsistent, RDSR values

At least one system appeared to sum irradiation events from different x‐ray tubes (fluoroscope and overhead radiography with different PERPs) into a single accumulated K_a,r_ value. Since K_a,r_ is distance‐dependent, this summation is fundamentally incorrect and can lead to dose misrepresentation. On another system, the summation of event‐level data, all with the same defined PERP, was found to not equal the reported values in the accumulated data. In a third system, the irradiation duration for fluoroscopy was inconsistent with the pulse rate and pulse width.

#### 3.1.4 Missing or zeroed dosimetry data

Some RDSRs lacked essential dose indices. On one system, P_KA_ values for all fluoroscopy events were zero. Another reported dose indices only for radiographic acquisition events. In multiple instances, zero values were reported for K_a,r_, P_KA_, kVp, and tube current, even though the information was available in alternative dose report formats. Further, some RDSRs reported non‐zero accumulated radiographic acquisition P_KA_ for studies that contained only fluoroscopic irradiation events.

#### 3.1.5 Identification of x‐ray source

The x‐ray tube responsible for each irradiation event in the RDSR was not well defined. For some systems, this could be inferred from the PERP or similar value, but many systems lacked the event‐level detail to differentiate the source of each irradiation event. Additionally, both radiographic acquisitions and overhead radiography exposures were combined in the accumulated “Acquisition” metrics.

## 4 DISCUSSION

The survey data among pilot participants provides insight into current and evolving technologies in health systems. The wide range of fluoroscope capabilities and facility practice differences add complexity to the development of the DIR for benchmarking diagnostic fluoroscopy procedures. Additionally, inconsistencies in RDSR contents results in further challenges to benchmarking efforts.

### 4.1 Patient exposure reference point

As expected, most of the fluoroscopes in the pilot used for traditional diagnostic fluoroscopy procedures were tube‐under‐table units (Table 2). However, approximately 20% of the fluoroscopes surveyed were C‐arm or tube‐over‐table units, which adds to the complexity of any DIR effort aimed at understanding typical exposure levels used in diagnostic fluoroscopy procedures. Each type of fluoroscope—tube‐over‐table, tube‐under‐table, fixed C‐arm, and mobile C‐arm—has a unique PERP based on the geometry of the system. The PERP is the location between the x‐ray focal spot and the detector where K_a,r_ and K_a,r_ rate are reported, both to the operator at the fluoroscope and typically within the RDSR, although there are exceptions as discussed later. The location of the PERP is set by FDA regulations and IEC standards.^8^, ^9^ The PERP for tube‐under‐table systems is 1 cm above the tabletop; for tube‐over‐table systems, the PERP is 30 cm above the tabletop; for fixed C‐arm systems, the PERP is 15 cm from isocenter towards the x‐ray tube; and for mobile C‐arms without cone beam CT capability, the PERP is 30 cm from the image receptor. Additionally, tube‐under‐table systems often utilize an additional overhead radiography x‐ray tube which can be used for additional imaging in diagnostic fluoroscopy procedures. For these x‐ray tubes, the reference point is often 30 cm above the tabletop. Vendors are allowed to specify alternative PERPs for C‐arm fluoroscopes without an isocenter and for certain fluoroscopes where the IEC standard does not specify a location for the PERP.^10^ All the units surveyed in this study used the standard PERP definitions for displayed dose indices; however, as discussed later, this was not true for dose indices reported within RDSRs.

The differences in PERP location create a challenge for using point‐measurements like K_a,r_ and K_a,r_ rate for benchmarking. Air Kerma decreases with distance from the focal spot of the x‐ray tube. Tube‐under‐table fluoroscopy systems have a reference point that corresponds approximately to the entrance surface of the patient. Meanwhile, for tube‐over‐table systems as well as for overhead radiography tubes in tube‐under‐table systems, the PERP is a fixed distance from the tabletop, and thus, the relationship of K_a,r_ and K_a,r_ rate to actual patient entrance exposure is dependent on patient size. Fixed C‐arms typically have a PERP defined based on the isocenter location and often utilize a floating table; this creates a more nuanced situation of entrance exposures being dependent on the table height, which itself is dependent on a combination of patient size, procedure needs, and operator preference. Mobile C‐arms also are subject to positioning differences for similar reasons as fixed C‐arms due to table position but utilize a different PERP than their fixed C‐arm counterparts. Interpretation of K_a,r_ measurements from mobile and fixed c‐arm fluoroscopes, including multipurpose c‐arm fluoroscopes in this study, is also complicated by the effect of patient table attenuation on patient exposure. Table attenuation depends on the positioning of the table and gantry.

All of these circumstances result in K_a,r_ values that may not be comparable across different types of fluoroscopes. Even values within a single procedure that utilizes both tube‐under‐table fluoroscope and an overhead radiography tube result in a mixture of K_a,r_ values that cannot be easily combined. Thus, while the interventional fluoroscopy DIR can largely utilize K_a,r_ as a primary metric due to relatively universal use of fixed C‐arm systems and therefore a consistent PERP definition, it is less useful for benchmarking for diagnostic procedures due to the substantial differences in PERP locations.

### 4.2 System age and technological capability

Approximately 62% of fluoroscopes surveyed incorporated FPDs, which indicates the increasing adoption of FPDs in diagnostic fluoroscopy areas, similar to the trends seen in Interventional Radiology. The availability of pulsed fluoroscopy on systems was nearly universal among surveyed systems.

The age distribution of the surveyed fluoroscopes at the time of the pilot (2022–2023) as well as the observed technological differences further complicate reporting efforts and the development of metrics such as Diagnostic Reference Levels (DRLs). Among the participating sites, the average age of fluoroscopes in the study was approximately 9.6 years with a large standard deviation (5.2 years). Fluoroscopes as old as 19 years were part of the surveyed data. This age distribution is likely to be similar to U.S. practice at large, suggesting that long service lifetimes, often beyond vendor end‐of‐life declarations, are not uncommon in diagnostic fluoroscopy. Some of the surveyed systems in operation lacked one or more commonly displayed dose indices (K_a,r_. K_a,r_ rate, or P_KA_), which can limit the ability for facility dose monitoring in any capacity. As seen in Figure 2, the system ages were strongly correlated with both detector technology (FPD vs II) and RDSR capability, with a major shift in both occurring in the early 2010s. Only 64% of surveyed units could produce RDSRs. This dual shift in technology has important implications for the Fluoroscopy DIR. Because the DIR relies entirely on RDSRs for dosimetry information, any benchmarking data or resultant DRLs will naturally be skewed heavily to newer systems equipped with FPDs. Facilities with older equipment, especially those with IIs, will thus be limited in what conclusions they can draw about typical dose levels in their diagnostic fluoroscopy procedures. Over time, this will be less of a concern as more systems are installed or replaced with RDSR capabilities. The DIR is also exploring ways of identifying detector technologies, which may allow for improved benchmarking for individual facilities. Until older units are replaced, any benchmarking using the DIR will primarily reflect the performance of modern, RDSR‐capable systems.

### 4.3 Dosimetric performance

Dosimetric accuracy was tracked for a 2‐year period of the pilot on a semi‐annual basis. Both fluoroscopic and radiographic acquisition dose accuracy of fluoroscopy x‐ray tubes were evaluated for displayed K_a,r_ and P_KA_ for 56 of the surveyed systems. Dose measurements were conducted regardless of RDSR capability, but analyses were conducted for both the entire dataset and for only RDSR capable systems given the requirements of DIR participation. As shown in Figure 3, the dosimetric deviations were largely centered around 0 with a relatively narrow bias towards positive values. This was expected, as even 25 years ago dosimetric output from fluoroscopes tended to be stable over time.^11^ Only one system tested outside the allowed ± 35% accuracy specified by the FDA and IEC;^9^, ^10^ this was subsequently resolved by servicing the system. Performance across fluoroscopy and radiographic acquisition modes, including both K_a,r_, and P_KA_, was largely similar. Linear mixed effect models were used to examine the impact of time and technology on the data to understand whether the deviations were stable over time, even after accounting for technological differences. From the results, time was found to not be significant for any of the best fit models nor the simplest model for RDSR‐capable units (Table 6), providing evidence that the dosimetric accuracy for systems tend to be stable over time for those units able to send dosimetry data to the DIR. Thus, the data from the pilot period is expected to be stable for the purposes of analyzing K_a,r_ and P_KA_ for diagnostic fluoroscopy procedures. Time was found to be significant for Rad Acquisition P_KA_ when including all surveyed units, but that result as well as general low *p*‐values of the P_KA_ measurements may be due to the sensitivity of P_KA_ accuracy to errors in user field size measurements. While the number of datapoints per system was limited during the study (up to 4), the results coupled with the observed deviations (Figure 3) support the conclusion that system drift is not significant over the studied time period. Moreover, data from the participants outside of the pilot group are likely to demonstrate comparable stability without the need for routine updates of dosimetry correction factors provided at least the minimum annual physics evaluations are performed as part of routine quality assurance. The data in this study were collected over a 2‐year period, which may not be adequate to capture long term drift characteristics. However, such drift should be observed and corrected during routine medical physics evaluations.

### 4.4 RDSR limitations

Perhaps the most important lesson from the pilot effort is a cautionary tale for institutions relying on RDSR data for diagnostic fluoroscopy.

One important component of the diagnostic fluoroscopy DIR pilot was to review RDSR data across the various fluoroscopes to ensure RDSR reliability. RDSRs for interventional fluoroscopes have been widely available since the 2010s, but the adoption of RDSR capability in other types of fluoroscopes has been far slower. This slower implementation is likely due in part to the perceived greater importance of RDSR data for higher dose clinical procedures and peak skin dose calculations. Due to this and the complexity of harnessing and combining dose data from tube‐under‐table, tube‐over‐table, fixed and mobile C‐arms, and overhead radiographic tubes, the pilot effort included a review of RDSRs from participating systems to identify unexpected challenges.

We identified several notable issues that impact both the DIR pilot effort as well as the dose monitoring efforts of any institution. These issues all impact tube‐under‐table fluoroscopes across multiple vendors and models and persist despite the current DICOM standard for RDSR implementation.^12^

First is the inconsistent identification and labeling of the PERP within the RDSR. As previously mentioned, the reference point is the location of the displayed air kerma. For tube‐under‐table fluoroscopes, the PERP is defined by the FDA and IEC as 1 cm above the tabletop. For several systems evaluated in this pilot, irregularities were identified in the RDSR's listed reference point. These issues were seen in tube‐under‐table systems, including listing multiple reference points for a single dose index or providing illogical reference points such a definition based on isocenter for a system that has no isocenter. Inclusion of 2 or 3 separate locations makes the accumulated K_a,r_ data unusable due to its dependence on reporting location. For the tube‐under‐table system reporting a K_a,r_ value at a location based on a non‐existent isocenter renders the data useless for comparison with other systems. Further challenges arise when systems deviate from the conventional reference point for tube‐under‐table systems (such as 30 cm above tabletop seen in some RDSRs for fluoroscopic exposures). Alternative PERP definitions make the accumulated data not directly comparable to traditional reference point data for these systems even if the provided reference points are accurate. Many of these reference point definition issues also persist in the individual irradiation event data. Such inconsistencies make it impossible to compare K_a,r_ values confidently across systems.

Many event level issues were identified. This included an inability to distinguish the x‐ray tube used in each irradiation event. While biplane fixed C‐arms utilize a “Plane A” and “Plane B” designation, no such identification was found for tube‐under‐table fluoroscopes and overhead radiography tubes. Furthermore, discrepancies in how event level data are summed into the accumulated data fields also presents a problem for benchmarking. The summing of K_a,r_ without regard for PERP differences was observed in some RDSRs. In others, several accumulated dose indices did not match the summation of event level data. These suggest inconsistent treatment of exposure values from different x‐ray tubes as well as underlying issues with how accumulated indices are calculated. The uncertainty of how events are summed creates a situation where event‐level data cannot be trusted to overcome some of the uncertainties in PERP definitions for the accumulated fields. Of note, the continued expansion of modern, large FPD in tube‐under‐table systems may help mitigate some of these issues by allowing for more radiographic exposures to be acquired using only the fluoroscope.

Potentially worse still is the observation that some systems do not report all dose indices for each irradiation event. As noted previously, one system recorded all fluoroscopy P_KA_ values were as 0 within the RDSR. On another, no dose indices were recorded for any fluoroscopy events—only radiographic events were recorded as irradiation events. Among those acquisition events, we commonly observed K_a,r_, P_KA_, kVp, x‐ray tube current, and other indices recorded as zero values even when the data were available in non‐RDSR dose report formats. Yet still on other studies that involved only fluoroscopic events, some RDSRs were found to contain non‐zero radiographic P_KA_ values.

Failure to report all irradiation events within the RDSR creates an environment where individual events cannot reliably be analyzed, summed, or, in the case of multiple x‐ray tubes, separated by exposure source. Even for systems that do reliably report every irradiation event, the introduction of PERP uncertainty at that level, including inability to distinguish between x‐ray tubes, makes distance‐dependent indices unreliable.

The large range of issues found across vendors and models added a level of complexity that was unexpected. Indices that depend on well‐defined locations, such as K_a,r_, must be considered unreliable for dose monitoring across the wide range of fluoroscopes that are used for diagnostic fluoroscopy procedures. This is due to both the inherently different reference points across tube‐under‐table, tube‐over‐table, and C‐arm geometries discussed earlier as well as the inconsistencies in PERP definitions within the RDSRs of the same geometry.

Thus, the DIR pilot is moving forward with a focus on P_KA_ as the primary dose index for diagnostic fluoroscopy procedures. P_KA_ has several inherent advantages in this environment—it is distance independent, which makes it better suited for comparing exposures across fluoroscopic geometries. It also lends itself to being used in conjunction with overhead radiographic P_KA_, as it can be summed as an index for exposure level although the exposure geometry and orientation is typically different from that for the fluoroscope. This is important because in many RDSRs used for tube‐under‐table systems, it may not be possible to distinguish whether irradiation events are from the fluoroscope or the radiographic x‐ray tube. P_KA_ is not only easier to work with across system types, but also more reliable as a comparative dose index in the absence of reliable PERP data. There is also the consideration that patient radiation doses are typically lower for diagnostic fluoroscopy than for interventional procedures, so tissue reactions are not a concern and K_a,r_ is of less interest clinically than P_KA_.^13^, ^14^ However, the risk of stochastic effects remains a potential concern. PKA is a better indicator of the total x‐ray energy imparted to the patient, and thus a better index for stochastic risks.

Our findings have implications for institutional dose monitoring as well. Great care should be taken to ensure that any dose monitoring for non‐interventional fluoroscopes is performed in a manner that is consistent with the data. Medical physicists are needed to verify dose index accuracy, identify discrepancies between displayed values and RDSR values, and carefully review both the accumulated and irradiation event level data to account for any discrepancies found in RDSR data. We recommend that users focus on P_KA_ as the primary dose index for any institutional benchmarking, both internally and externally, especially if the institution or health system utilizes various vendors or models of fluoroscopes.

## 5 CONCLUSION

The survey data and performance monitoring of multiple fluoroscopes participating in the pilot for the ACR's Diagnostic Fluoroscopy Dose Index Registry provide important insights into both typical technological practice standards and dosimetric performance. This multi‐institution study provides key insights into the range of fluoroscopy technologies used in diagnostic practice and demonstrates the overall stability of key dose indices across a 2‐year period. The stability of dose measurements should allow robust levels of benchmarking to be conducted through the DIR program, including the establishment of DRLs that reflect current practice, even for less commonly performed exams, thanks to the expected scale of participation.

However, our investigation of RDSR inconsistencies yielded important findings for both the DIR and individual facilities. A lack of standardization or compliance with dose reporting requirements, especially with regards to the location of reference points and the handling of irradiation events from multiple x‐ray tubes, creates challenges for dose monitoring at all levels. Additional problems, such as missing data for some irradiation events, RDSR fields left blank or set to 0, and mistakenly populated RDSR dose values such as non‐zero radiography P_KA_ when no radiographic images are acquired further complicates meaningful comparisons and benchmarking. Thus, we recommend that facilities engaged in dose monitoring of diagnostic fluoroscopy procedures utilize distance‐independent dose indices such as P_KA_ as the primary quantity of interest unless a medical physicist can verify the reporting accuracy of each of their diagnostic fluoroscopes within the RDSR through a detailed investigation of how each system reports accumulated and irradiation‐event level dose indices. Much like the errors in RDSRs in interventional fluoroscopes that were addressed over time with increased clinical adoption, we hope that over time vendors improve the consistency of RDSR implementation in non‐interventional fluoroscopes to remove uncertainty and ambiguity in dose reporting.

## AUTHOR CONTRIBUTIONS

All authors were participants in the ACR's Dose Index Registry Diagnostic Fluoroscopy pilot. All authors (except for Donald Miller) acquired technical and performance data for systems at their facilities across the pilot period. All authors engaged in review and discussion of data, including RDSR contents and discrepancies, and contributed to the writing and editing of the manuscript. Donald Miller also served as an invaluable clinical consultant and played a strong role in preparing the manuscript.

## CONFLICT OF INTEREST STATEMENT

The authors declare no conflicts of interest.
